# Open Reduction of an Isolated Anterior Nasal Spine Fracture: A Case Report and Review of the Literature

**DOI:** 10.1055/a-2107-2071

**Published:** 2023-08-02

**Authors:** Jinwoo Park, In Sik Yun, Tai Suk Roh, Young Seok Kim

**Affiliations:** 1 Department of Plastic and Reconstructive Surgery, Gangnam Severance Hospital, Yonsei University College of Medicine, Seoul, Korea

**Keywords:** open reduction, anterior nasal spine fracture, isolated anterior nasal spine fracture

## Abstract

A 14-year-old girl had a midfacial trauma event caused by hitting against an opening door and experienced discomfort and swelling of the columella and upper lip. Physical examination revealed mild tenderness on light palpation without any discomfort with upper lip movement. A computed tomography scan of the maxillofacial bones with three-dimensional reconstruction showed a fracture of the anterior nasal spine with obvious leftward displacement, mild-deviation of the caudal aspect of the nasal septum, and no sign of nasal bone fracture. Open reduction and internal fixation was performed with regard to aesthetic and functional concerns, including nasal septum deviation. The postoperative course was uneventful, and healing proceeded normally without complications. Herein, we emphasize the importance of differential diagnosis of isolated anterior nasal spine fractures in patients with midfacial trauma and clinicians' strategic decision-making in treatment modalities.

## Introduction

Anterior nasal spine (ANS) fracture, a rare type of midfacial fracture, was first documented in 1979. 1 Most patients with ANS fracture have been managed conservatively in the literature, and there is only one report of open reduction and internal fixation (ORIF) of ANS fractures. 2 In this case report, we describe another case of ANS fracture with severe displacement that was treated with ORIF. The patient's legal guardian provided written informed consent for the publication and the use of the patient's images.

## Case

**Fig. 1:**
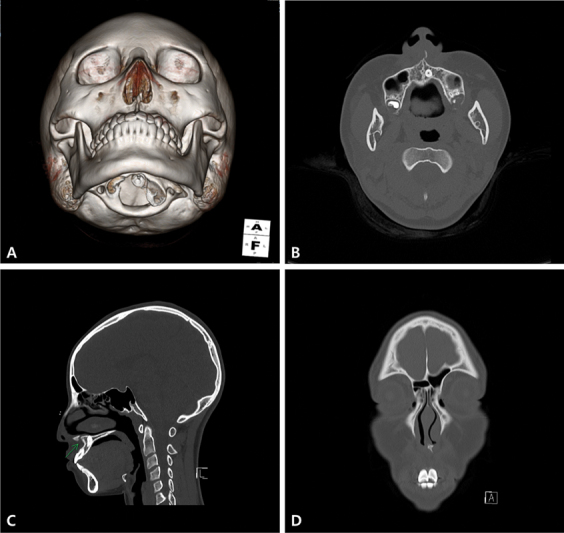
Preoperative computed tomography (CT) scan with three-dimensional (3D) reconstructions. Note that the anterior nasal spine was fractured and displaced to the left. ( **A** ) A worm's eye view in 3D reconstructed CT. ( **B** ) Axial CT image. ( **C** ) Sagittal CT image. ( **D** ) Coronal CT image.

A 14-year-old girl injured her face by hitting against an opening door. She immediately experienced pain and swelling of the columella and upper lip. She visited a local plastic surgery clinic and was suspected to have an ANS fracture. She was referred to our outpatient clinic 8 days after the trauma event. At presentation, she had no specific complaints except for mild swelling of the upper lip. Physical examination revealed mild tenderness on light palpation of the columella, without any discomfort with upper lip movement. A computed tomography (CT) scan of the maxillofacial bones with three-dimensional (3D) reconstruction revealed a fracture of the ANS with severe leftward displacement, mild deviation of the caudal aspect of the nasal septum, and no sign of nasal bone fracture ( Fig. 1 ).

**Fig. 2:**
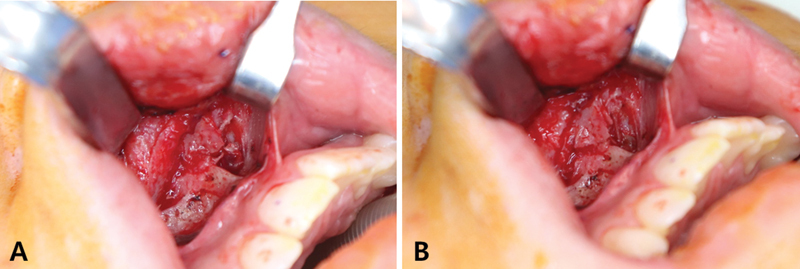
Intraoperative photograph before internal fixation. ( **A** ) A displacement of the anterior nasal spine to the left side was noted. ( **B** ) The anterior nasal spine is located at the original position after manual reduction.

**Fig. 3:**
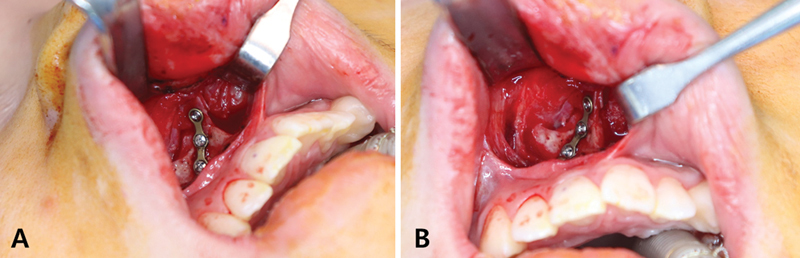
Intraoperative photograph after internal fixation. ( **A** ) Lateral view. ( **B** ) Frontal view.

ORIF of the ANS fracture was planned to address further functional and aesthetic concerns. Under general endotracheal anesthesia, the patient was placed in a supine position on the operative table. After the local anesthetic injection, a right upper buccal sulcus incision was made, and the dissection deepened into the subperiosteal layer, exposing the ANS. Displacement of the ANS on the left side was observed ( Fig. 2 ). The ANS fragment was gently handled and placed in its original position. After the position was confirmed, a three-hole microplate made of a four-hole microplate was bent adequately and the bone fragment was fixed to the remnant portion of the ANS with three 4-mm screws ( Fig. 3 ). The incision was repaired with absorbable sutures. The patient tolerated the procedure well without any intraoperative complications. The operative time was 52 minutes, and the estimated blood loss was minimal.

Postoperatively, the patient reported only mild pain in the columellar area and was discharged the day after surgery. The patient was instructed to gargle regularly, eat a relatively soft diet, and avoid excessive lip manipulation for a couple of weeks. At the postoperative visit to the outpatient clinic 2 weeks after surgery, the patient showed complete relief of symptoms, and the incision wound had healed well without complications.

## Discussion

The ANS is a small bony tubercle located at the lower edge of the piriform aperture. It is surrounded by relatively protruding adjacent facial structures, such as the nasal bone, nasal cartridge, malar bones, mandible, maxilla, and maximum teeth. 3 Concerning this unique anatomical structure and location, the ANS is regarded as less exposed to trauma and not easily fractured, unlike the nasal bone, which is most frequently fractured in facial injuries. 1 4 5 6 On the other hand, the actual incidence of ANS fracture may not be as low as predicted. You et al 7 recently reported that the rate of ANS fracture is 22% and that the diagnosis was missed in 95% of 200 cases of axial CT images performed for maxillofacial trauma in a single hospital.

Therefore, the differential diagnosis of ANS fractures should be considered in patients with midfacial trauma. The common clinical symptoms of ANS fractures are swelling, tenderness, mouth bleeding, mucosal lip laceration, and submucosal ecchymosis. 1 2 3 4 6 These clinical symptoms can be ignored by patients, and clinicians may overlook physical examinations and radiological findings. 2 3 Our patient in this study showed only swelling and tenderness at the nasolabial angle, and we could confirm ANS fracture only after radiologic findings of dislocated ANS on CT. A maxillofacial CT scan is the preferred imaging modality for nasal bone fractures 8 and is also the most accurate diagnostic tool for ANS fractures. 6 Kim et al 2 also reported a case of ANS fracture that was not identified in the simple nasal bone and skull X-ray series but was revealed only in the facial bone CT with 3D reconstruction.

According to the recent systematic review of Raggio et al, 6 there is no definitive recommendation regarding the management of ANS fracture. Most ANS fractures in previous literature had mild or no displacement of the ANS, and conservative treatments such as analgesia, oral decongestants, ice packs, antibiotics, and determined lip manipulation were performed to control symptoms. 1 3 4 5 6 9 However, considering basic principles of facial trauma, conservative treatments only would have a chance of possible adverse events such as floating or nonunion of bone fragments, while surgical intervention would help in assuring a successful recovery of bony continuity. In the case of severe displacement of the ANS with a symptom of ambiguous pain over the nasolabial angle exposed by upper lip closure movement, 2 ORIF was performed to provide bony stability and prevent the ANS from floating or nonunion.

Our patient was a young teen without distinct symptoms, and we performed ORIF mainly considering the aesthetic concerns. It is well known in previous literature that the repositioning of the ANS is correlated with cosmesis. Deviated ANS is associated with asymmetric nostrils, slanted columella, asymmetric and blunted nasal tips, and nasal airway obstruction, and is a common cause of caudal septal deviation. 10 Deformation of the ANS can cause various imperfections in the lower third of the nasal pyramid; therefore, reshaping of the ANS during rhinoplasty should not be overlooked. 11 ANS relocation for deviated ANS may improve aesthetic and functional outcomes in cleft orthognathic surgery in patients with unilateral cleft nasal deformity. 12

There is also functional dysfunction due to a deviated ANS because of the relationship between the septal cartilage and ANS. Correction of the impaired caudal septal ANS connection is imperative for opening the air passage in some cases of nasal septal deviation. 13 In our patient, mild deviation of the caudal aspect of the septal cartilage was observed on maxillofacial CT. Although she had no distinct respiratory symptoms, including difficulty breathing, she reported mild nasal obstruction before the trauma event. Therefore, we expected that surgical intervention may prevent further aggravation of nasal obstruction.

In our decision-making process, we carefully evaluated the necessity of performing the invasive surgical intervention and considered the potential disadvantages. The most common complications about fixation after titanium plates and screws in patients with traumatic facial fractures are known as discomfort related to palpability, cold intolerance, and pain. 14 Intraoral incision during procedures could lead to a postoperative wound infection, which is the most common postoperative complications in head and neck surgeries. 15 These complications often require secondary surgical procedures for hardware removal. In addition, unnecessary medical expenses and required hospital stays after general anesthesia should be taken into consideration. To reduce the risk of these complications, we used a three-hole microplate for fixation, administered prophylactic antibiotics pre- and postoperatively, and minimized the hospital stay.

We considered the various aspects described above to determine the treatment approach and eventually decided to perform ORIF. Given the patient's age of 14 years, the completion of her facial growth had been achieved and she would benefit from surgical correction since in girls the midfacial height and projection mature at 12 and 13 years of age, respectively. 16 We concluded that the possibility of later development of facial asymmetry and worsening of nasal obstruction due to a deviated ANS was significant and that the benefit of corrective surgical intervention outweighed the potential postoperative complications. Furthermore, closed reduction of the ANS fracture could have been considered, but due to the severe displacement of the ANS, we performed the internal fixation of the bone fragment in order to ensure definitive correction of the ANS. Our decision was also supported by previous literature, 6 which did not identify any cases of ANS correction by closed reduction.

In conclusion, we performed ORIF with microplate for a patient with isolated ANS fracture. The surgical technique itself was not innovative, but we aimed to address ORIF as one of the treatment options of ANS fracture for various reasons. We also suggest that clinicians always keep in mind the possibility of isolated ANS fractures in patients with midfacial trauma. Taking a maxillofacial CT scan with 3D reconstruction would help avoid missed diagnoses. The modality of treatment including open reduction should be carefully considered with regard to the severity of the fracture, functional symptoms, and aesthetic consequences.
